# Glenohumeral internal rotation deficit after operative treatment of mid-shaft clavicle fractures: a subclinical, scapula-related phenomenon

**DOI:** 10.1186/s13018-026-06856-7

**Published:** 2026-04-24

**Authors:** QingLyu Shi, RunZe Li, Hua Li, LiChao Zhang, YanLin Li, GuoLiang Wang

**Affiliations:** The Department of Sports Medicine, the First Affiliated Hospital of Kunming Medical University, No.295 Xichang Road, Wuhua District, Kunming, 650032 China

**Keywords:** Glenohumeral internal rotation deficit, Scapular protraction, Mid-shaft clavicle fractures, Open reduction and internal fixation, Rehabilitation strategies

## Abstract

**Background:**

Glenohumeral internal rotation deficit (GIRD) is a well-recognized component of shoulder dysfunction and has been extensively described in overhead athletes within the framework of scapular dyskinesis. However, whether a similar or distinct pattern of GIRD exists after mid-shaft clavicle fractures remains insufficiently understood. This study aimed to evaluate the prevalence and features of GIRD in a post-traumatic population and to explore their potential relationship with scapular positioning.

**Methods:**

This cross-sectional comparative study included 99 patients with healed mid-shaft clavicle fractures, comprising 62 patients treated with open reduction and internal fixation (ORIF) and 37 patients managed conservatively. Internal and external rotation were measured bilaterally using a standardized supine protocol, and side-to-side differences were calculated to identify GIRD. Scapular dyskinesis was assessed using the SICK Scapula Rating Scale, and subjective function was evaluated using the Disabilities of the Arm, Shoulder and Hand (DASH) score. Scapular orientation was indirectly and exploratorily assessed on standardized anteroposterior radiographs using group-specific projected area ratios. Within the ORIF cohort, exploratory analyses were also performed to identify factors potentially associated with postoperative GIRD.

**Results:**

The ORIF group demonstrated a significantly greater internal rotation deficit than the conservative group (25° [11.5, 27] vs. 0° [0, 5], *P* < 0.001). No significant difference in external rotation gain was observed between groups. The ORIF group also showed a significantly greater G-C ratio and higher SICK Scapula Rating Scale scores than the conservative group, while DASH scores remained similarly low in both groups. Within the ORIF cohort, 45 of 62 patients (72.6%) met the predefined criterion for GIRD. Compared with patients without GIRD, those with GIRD demonstrated significantly higher values for both the G-G ratio and the G-C ratio as well as higher SICK Scapula Rating Scale scores, but no significant difference in DASH scores. No routinely recorded demographic, injury-related, or peri-treatment variable was significantly associated with postoperative GIRD in the ORIF group.

**Conclusion:**

GIRD appears to be a common but largely subclinical finding after operative treatment of mid-shaft clavicle fractures. In contrast to the pattern described in overhead athletes, this post-traumatic internal rotation deficit was not accompanied by external rotation gain, suggesting a potentially distinct underlying mechanism. The observed association with a tendency toward scapular protraction may provide insight into this phenomenon. Awareness of this pattern may be relevant to postoperative assessment and may help inform future studies aimed at optimizing rehabilitation strategies.

**Supplementary Information:**

The online version contains supplementary material available at 10.1186/s13018-026-06856-7.

## Background

Clavicle fractures are common injuries in orthopedic trauma, accounting for approximately 2.6–4% of all skeletal fractures [1]. Among these, displaced mid-shaft clavicle fractures represent the majority of cases [2]. Although nonoperative treatment was historically considered the standard of care [3], surgical management has gained increasing acceptance in recent years [4]. This shift has been driven by patient expectations for anatomical restoration, earlier functional recovery, and a timely return to daily activities. Open reduction and internal fixation (ORIF) offers reliable fracture stabilization and generally facilitates early mobilization [5].

Despite successful restoration of bony alignment, postoperative rehabilitation protocols following clavicle fracture fixation have primarily focused on fracture protection and subsequent glenohumeral mobilization [6]. Comparatively less attention has been directed toward the potential influence of trauma, surgical exposure, and early immobilization on scapulothoracic mechanics. The clavicle functions as the only osseous connection between the upper extremity and the axial skeleton and plays an essential role in maintaining both static and dynamic scapular stability [7]. As such, alterations in postoperative biomechanics may influence scapular kinematics beyond what is captured by fracture healing alone [8, 9].

Within the framework of shoulder biomechanics, scapular dyskinesis has been recognized as an important contributor to altered shoulder function. Kibler and colleagues introduced the concept of the ‘risk shoulder’, emphasizing that subtle, often asymptomatic abnormalities—such as scapular protraction [10] and glenohumeral internal rotation deficit (GIRD) [11]—may predispose the shoulder to dysfunction over time. In overhead athletes, GIRD has been extensively described as a key component of a pathological cascade associated with posterior capsular tightness and altered shoulder mechanics, and is often accompanied by a compensatory gain in external rotation [11]. However, whether a similar or distinct pattern of GIRD exists in post-traumatic populations remains insufficiently defined [12].

Importantly, commonly used patient-reported outcome measures (PROMs), including the Disabilities of the Arm, Shoulder and Hand (DASH) score, are designed to detect perceived functional impairment [13] and may be less sensitive to subclinical kinematic alterations. As a result, patients who report satisfactory outcomes after fracture union may still exhibit objective biomechanical deviations that are not readily apparent during routine clinical follow-up.

Accordingly, the present study aimed to investigate glenohumeral internal rotation and scapular orientation after union of mid-shaft clavicle fractures in patients treated either operatively or conservatively. Specifically, we sought to (1) determine the prevalence of clinically relevant GIRD in this post-traumatic cohort, (2) examine the relationship between objective kinematic findings and patient-reported outcomes, and (3) explore the potential utility of standard anteroposterior radiographs and projected area ratios as indirect and exploratory markers of altered scapular positioning.

## Methods

### Patients

This cross-sectional comparative study included patients with healed mid-shaft clavicle fractures who were treated at our institution. The operative group consisted of 62 patients who had undergone ORIF and were assessed at the time of elective implant removal between January 2023 and December 2024. The conservative group consisted of 37 patients with mid-shaft clavicle fractures managed nonoperatively who underwent clinical follow-up evaluation after fracture union. All patients had achieved clinical union at the time of assessment. Inclusion criteria were defined to ensure a relatively homogeneous cohort of healed mid-shaft clavicle fractures. Exclusion criteria were as follows: (1) revision surgery; (2) concomitant fractures of the ipsilateral upper extremity; and (3) pre-existing comorbidities affecting shoulder function, such as inflammatory arthritis or neurological disorders. Because treatment allocation was based on clinical decision-making rather than randomization, the operative and conservative groups were not intended to be fully matched, and comparative analyses were interpreted in this context.

### Clinical assessment

**Fig. 1:**
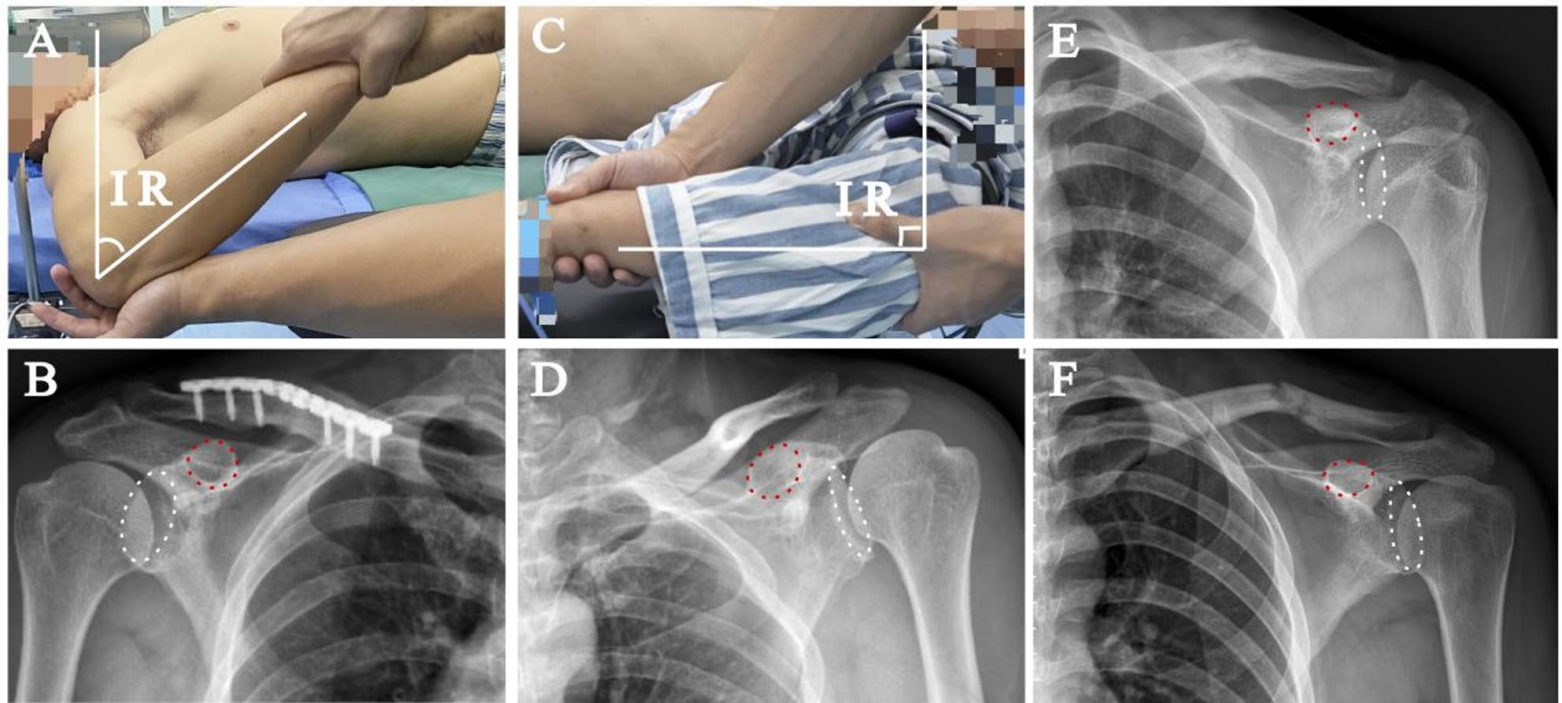
Clinical and radiographic assessment of glenohumeral internal rotation and projected area measurements.**A**, **C** Glenohumeral internal rotation (IR) measured in the supine position with the shoulder abducted to 90° and the elbow flexed to 90°. **B**, **D** Standardized anteroposterior (AP) shoulder radiographs from a patient in the ORIF group showing bilateral glenoid projected area measurement; the affected side is shown in (**B**) (plate fixation visible), and the contralateral healthy side in (**D**). **E**, **F** Standardized AP shoulder radiographs illustrating measurement of the projected glenoid surface area and coracoid process area for calculation of the ipsilateral glenoid-to-coracoid projected area ratio. The white dotted outline indicates the projected glenoid surface area, and the red dotted outline indicates the projected coracoid process area

Clinical evaluation was performed in both groups with a focus on GIRD and scapular kinematics. (1) Range of Motion (ROM): Assessment was conducted with the patient in a supine position to stabilize the scapula. The humerus was abducted to 90° with the elbow flexed to 90°. Internal and external rotation angles were measured using a standard goniometer (Fig. 1A, C). Measurements were taken bilaterally to allow for comparison between the affected and contralateral healthy sides. GIRD was defined as a side-to-side difference of 20° or greater, in accordance with criteria previously described by Kibler and colleagues [11]. (2) Scapular Dyskinesis: Scapular dyskinesis was assessed using the SICK (scapular malposition, inferomedial border prominence, coracoid pain and malposition, and dyskinesis of scapular movement) Scapula Rating Scale described by Kibler et al. [10]. (3) Functional Outcome: The Disabilities of the Arm, Shoulder and Hand (DASH) [13] questionnaire was administered to quantify subjective functional status.

In addition, potentially influential clinical variables, including dominant-side involvement, mechanism of injury, associated shoulder sensory symptoms, duration of sling use, fracture classification, postoperative wound complications, and rehabilitation compliance assessed by the Exercise Adherence Rating Scale (EARS) [14], were recorded for comparative analyses.

### Radiologic measurement

Standardized anteroposterior (AP) shoulder radiographs were obtained to evaluate scapular orientation. To ensure reproducibility and minimize projection error, a strict positioning protocol was implemented: patients stood upright with their head, back, buttocks, and heels maintaining contact with the detector panel (DR receptor). Both arms hung naturally at the sides in neutral rotation.

On the digital radiograph, the projected surface areas of the glenoid fossa and coracoid process were measured (in mm²; Fig. 1B, D, E, F). Because contralateral paired radiographs were available in the ORIF cohort but not in the conservative cohort, different radiographic ratios were used according to the study subgroup. In the ORIF group, a bilateral glenoid projected area ratio (G-G ratio) was calculated as affected-side glenoid area divided by contralateral-side glenoid area. In addition, an ipsilateral glenoid-to-coracoid projected area ratio (G-C ratio) was calculated as glenoid area divided by coracoid area on the affected side. In the conservative group, scapular orientation was assessed using the G-C ratio only. These parameters were used as indirect and exploratory radiographic surrogates of scapular orientation rather than as direct measures of scapular protraction.

### Statistical analysis

All statistical analyses were performed using IBM SPSS Statistics 27.0 (IBM Corp., Armonk, NY, USA). The normality of continuous variables was assessed using the Kolmogorov-Smirnov test. Normally distributed data were expressed as mean ± standard deviation (SD) with 95% confidence intervals (CIs), while non-normally distributed data were presented as median with interquartile range (IQR, 25th–75th percentiles). For comparisons between the affected and contralateral sides, the paired-sample t-test was used for normally distributed data, and the Wilcoxon signed-rank test was used for non-normally distributed data. Between-group comparisons of continuous variables were performed using the Mann–Whitney U test. Comparisons of categorical variables were performed using the chi-square test or Fisher’s exact test, as appropriate. Subgroup analyses were performed within the ORIF cohort according to the presence or absence of GIRD. Radiographic comparisons between the ORIF and conservative groups were performed using the G-C ratio. Within the ORIF cohort, both the G-G ratio and the G-C ratio were analyzed in relation to GIRD status. A P value < 0.05 was considered statistically significant.

## Results

**Fig. 2:**
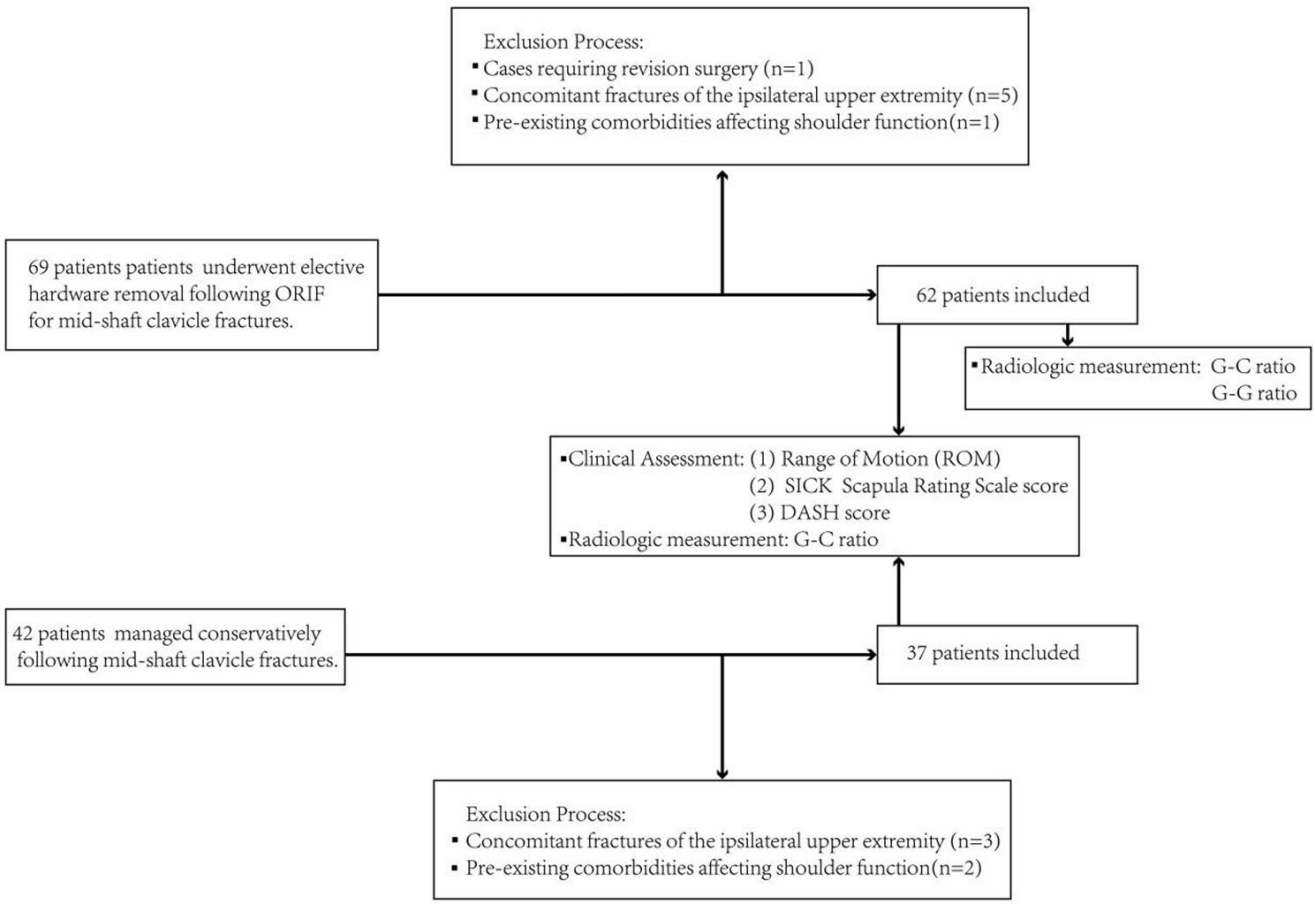
Flow diagram of patient inclusion, exclusion, and assessment in the operative and conservative cohorts. ORIF, open reduction and internal fixation; ROM, range of motion; SICK, scapular malposition, inferomedial border prominence, coracoid pain and malposition, and dyskinesis of scapular movement; DASH, Disabilities of the Arm, Shoulder and Hand; G-G ratio, affected-side glenoid projected area / contralateral-side glenoid projected area; G-C ratio, ipsilateral glenoid projected area / ipsilateral coracoid projected area

A total of 99 patients with mid-shaft clavicle fractures were included in the analysis, comprising 62 patients treated with ORIF and 37 patients managed conservatively. Figure 2 illustrates the detailed patient selection and exclusion process. The baseline characteristics of the two groups are summarized in Table 1. Patients in the ORIF group were older than those in the conservative treatment group (45.8 ± 6.8 vs. 22.4 ± 7.9 years). The sex distribution and affected side were comparable between groups. The mean follow-up duration was 16.8 ± 2.4 months in the ORIF group and 6.6 ± 1.9 months in the conservative group. Regarding fracture pattern, all patients in the ORIF group had Robinson type 2B fractures (2B1: *n* = 55; 2B2: *n* = 7), whereas all patients in the conservative group had Robinson type 2 A fractures (2A1: *n* = 32; 2A2: *n* = 5). Notably, regardless of the kinematic findings, the overall subjective recovery was excellent, as evidenced by a mean DASH score of 0.43 ± 0.75, indicating that the majority of patients perceived minimal to no disability in their daily activities.

**Table 1:** Baseline characteristics of the operative and conservative treatment groups

Variable	ORIF group (*n* = 62)	Conservative treatment group (*n* = 37)
Age, years	45.8 ± 6.8 (33 ~ 64)	22.4 ± 7.9 (15 ~ 40)
Sex (female/male), n	25/37	17/20
Affected side, left/right, n	20/42	14/23
Follow-up duration, months	16.8 ± 2.4 (14 ~ 22)	6.6 ± 1.9 (3 ~ 12)
Robinson classification, n		
2A1	0	32
2A2	0	5
2B1	55	0
2B2	7	0
DASH score	0.40 ± 0.76	0.50 ± 0.73
SICK scapula rating scale score	1.10 ± 0.92	0.27 ± 0.51

Data are presented as mean ± standard deviation (range) for continuous variables and as number for categorical variables. ORIF, open reduction and internal fixation; DASH, disabilities of the arm, shoulder and hand

Comparative analysis of kinematic, radiographic, and functional outcomes between the ORIF and conservative treatment groups is shown in Table 2. The ORIF group demonstrated a substantially greater GIRD than the conservative group (median 25° [11.5, 27] vs. 0° [0, 5], *P* < 0.001). Notably, no clinically relevant GIRD was identified in the conservative treatment group, whereas GIRD was common in the ORIF group. No significant difference in ER gain was observed between the two groups (median 0° [0, 0] in both groups, *P* = 0.154). The G-C ratio was significantly greater in the ORIF group than in the conservative group (2.12 [1.39, 2.47] vs. 1.04 [0.92, 1.13], *P* < 0.001). Although DASH scores were similarly low in both groups (*P* = 0.403), the ORIF group had significantly higher SICK Scapula Rating Scale scores than the conservative group (1 [0, 2] vs. 0 [0, 0.5], *P* < 0.001).

**Table 2:** Comparison of kinematic, radiographic, and functional outcomes between the operative and conservative treatment groups

Outcome measure	ORIF group (*n* = 62)	Conservative group (*n* = 37)	*P* value
IR deficit (°)^a^	25 [11.5, 27]	0 [0, 5]	< 0.001
ER gain (°)^b^	0 [0, 0]	0 [0, 0]	0.154
G-C ratio^c^	2.12 [1.39, 2.47]	1.04 [0.92, 1.13]	< 0.001
DASH score	0 [0, 1]	0 [0, 1]	0.403
SICK scapula rating scale score	1 [0, 2]	0 [0, 0.5]	< 0.001

Data are presented as median [25th, 75th percentiles] due to non-normal distribution. Statistical significance was determined using the Mann–Whitney U testIR, internal rotation; ER, external rotation; DASH, disabilities of the arm, shoulder and hand; ORIF, open reduction and internal fixation

^a^Calculated as (Contralateral IR − Affected IR)

^b^Calculated as (Affected ER − Contralateral ER)

^c^Calculated as (Ipsilateral glenoid projected area/ipsilateral coracoid projected area)

 Potentially influential clinical variables in the two treatment groups are presented in Table 3. Compared with the conservative treatment group, patients in the ORIF group were significantly older (45 [40, 49] vs. 20 [16, 25.5] years, *P* < 0.001) and had a longer follow-up duration (16 [15, 18] vs. 6 [6, 7.5] months, *P* < 0.001). The proportion of dominant-side injury was similar between groups (*P* = 0.814), as were the distributions of injury mechanism (direct vs. indirect trauma, *P* = 0.762) and associated shoulder sensory symptoms (*P* = 0.966). The duration of sling use was significantly shorter in the ORIF group than in the conservative group (4 [3, 4] vs. 6 [6, 6] months, *P* < 0.001), whereas rehabilitation compliance, as assessed by the EARS score, did not differ significantly between groups (*P* = 0.694).

**Table 3:** Comparison of baseline and potentially influential clinical variables between the operative and conservative treatment groups

Variable	ORIF Group (*n* = 62)	Conservative Group (*n* = 37)	*P* value
Age, years	45 [40, 49]	20 [16, 25.5]	< 0.001
Follow-up duration, months	16 [15, 18]	6 [6, 7.5]	< 0.001
Injury to dominant side, yes/no	40/22	23/14	0.814
Mechanism of injury, direct/indirect	9/53	4/33	0.762^a^
Associated nerve symptoms (shoulder numbness or hypoesthesia), yes/no	17/45	10/27	0.966
Duration of sling use, months	4 [3, 4]	6 [6, 6]	< 0.001
Rehabilitation compliance (EARS score)	20 [18, 24]	20 [17, 24]	0.694

Data are presented as median [25th, 75th percentiles] for continuous variables and as number for categorical variables. Group comparisons were performed using the Mann–Whitney U test, chi-square test, or Fisher’s exact test, as appropriate. ORIF, open reduction and internal fixation; EARS, exercise adherence rating scale

^a^Fisher’s exact test

Within the ORIF cohort, patients were stratified into two subgroups based on the presence of GIRD, defined as a side-to-side difference of ≥ 20°, as described above. This resulted in 45 patients (72.6%) in the GIRD group and 17 patients (27.4%) in the Non-GIRD group (Table 4). Within the ORIF cohort, both the G-G ratio and the G-C ratio were significantly higher in patients with GIRD than in those without GIRD (*P* < 0.001). Similarly, the SICK Scapula Rating Scale score was significantly higher in patients with GIRD (1 [1, 2] vs. 0 [0, 1], *P* < 0.001). However, DASH scores remained low and did not differ significantly between groups (*P* = 0.789). Furthermore, unlike the pattern typically observed in overhead athletes [15, 16], no ER Gain was observed in this post-traumatic cohort (median 0° difference in both groups).

**Table 4:** Comparison of kinematic, radiographic, and functional outcomes between patients with and without GIRD in the ORIF group

Outcome measure	GIRD group (*n* = 45)	Non-GIRD group (*n* = 17)	*P* value
IR deficit (°)^a^	25 [25, 28]	10 [0, 10]	< 0.001
ER gain (°)^b^	0 [0, 0]	0 [0, 0]	0.454
G-G ratio^c^	2.32 [2.13, 2.43]	1.09 [0.98, 1.16]	< 0.001
G-C ratio^d^	2.33 [2.03, 2.52]	0.96 [0.89, 1.32]	< 0.001
DASH score	0 [0, 1]	0 [0, 0.5]	0.789
SICK Scapula Rating Scale score	1 [1, 2]	0 [0, 1]	< 0.001

Data are presented as median [25th, 75th percentiles] due to non-normal distribution. Statistical significance was determined using the Mann–Whitney U testIR, internal rotation; ER, external rotation; DASH, disabilities of the arm, shoulder and hand; GIRD, glenohumeral internal rotation deficit

^a^Calculated as (Contralateral IR − Affected IR)

^b^Calculated as (Affected ER − Contralateral ER)

^c^Calculated as (Affected-side glenoid projected area / contralateral-side glenoid projected area)

^d^Calculated as (Ipsilateral glenoid projected area / ipsilateral coracoid projected area)

An exploratory analysis of potential factors associated with postoperative GIRD in the ORIF group is shown in Table 5. No significant differences were observed between the GIRD and Non-GIRD groups with respect to age (*P* = 0.710), follow-up duration (*P* = 0.943), dominant-side involvement (*P* = 0.242), mechanism of injury (*P* = 0.423), associated shoulder sensory symptoms (*P* = 0.524), fracture classification (*P* = 0.662), postoperative wound complications (*P* = 0.555), duration of sling use (*P* = 0.411), or rehabilitation compliance assessed by the EARS score (*P* = 0.358). Therefore, none of the routinely recorded demographic, injury-related, or peri-treatment variables examined in the present cohort was identified as a significant factor associated with the presence of postoperative GIRD after ORIF.

**Table 5:** Analysis of potential factors associated with postoperative GIRD in the ORIF group

Variable	GIRD group (*n* = 45)	Non-GIRD group (*n* = 17)	*P* value
Age, years	45 [42, 48]	44 [39, 51]	0.710
Follow-up duration, months	16 [14.5, 18.5]	17 [15, 18]	0.943
Injury to dominant side, yes/no	31/14	9/8	0.242
Mechanism of injury, direct/indirect	8/37	1/16	0.423^a^
Associated nerve symptoms (shoulder numbness or hypoesthesia), yes/no	11/34	6/11	0.524
Fracture classification, 2B1/2B2	39/6	16/1	0.662^a^
Postoperative wound complications, yes/no	3/42	0/17	0.555^a^
Duration of sling use, months	4 [3, 4]	4 [3, 4]	0.411
Rehabilitation compliance (EARS score)	20 [18, 24]	20 [17.5, 24]	0.358

Data are presented as median [25th, 75th percentiles] for continuous variables and as number for categorical variables. Group comparisons were performed using the Mann–Whitney U test, chi-square test, or Fisher’s exact test, as appropriate. ORIF, open reduction and internal fixation; GIRD, glenohumeral internal rotation deficit; EARS, exercise adherence rating scale

^a^Fisher’s exact test

## Discussion

The clavicle serves as an essential osseous strut for both static and dynamic scapular stability [8, 17, 18]. In the present study, an apparent dissociation was observed between subjective functional recovery and objective shoulder kinematics after union of mid-shaft clavicle fractures. Although patient-reported disability was minimal in both groups, clinically relevant GIRD was common in the ORIF group and was not identified in the conservatively treated group. In addition, patients with GIRD in the ORIF cohort demonstrated significantly higher values for both the G-G ratio and the G-C ratio, along with higher SICK Scapula Rating Scale scores, whereas DASH scores remained low and did not differ significantly according to GIRD status. Taken together, these findings suggest that post-traumatic GIRD after operative treatment may represent a largely subclinical, scapula-related phenomenon that is not adequately captured by conventional patient-reported outcome measures, and the underlying mechanism may differ from the posterior capsular tightness model typically described in throwing athletes by Kibler [10]. Although the long-term significance of such asymptomatic abnormalities cannot be established in a cross-sectional setting, altered scapular positioning combined with GIRD may affect shoulder mechanics during daily or athletic activities. From a biomechanical perspective, this pattern could modify the effective center of rotation of the shoulder joint and potentially influence load distribution across periarticular structures [19]. Whether such a latent mechanical environment contributes to later-stage shoulder dysfunction remains to be determined through longitudinal follow-up studies.

An important consideration in interpreting the present findings is that treatment allocation was based on clinical decision-making rather than randomization. Therefore, the operative and conservative groups were not fully comparable at baseline. Patients in the conservative group were younger and generally had less severe fracture patterns, both of which may have influenced postoperative shoulder kinematics. Accordingly, although no clinically relevant GIRD was identified in the conservative group, the observed between-group difference should not be interpreted as evidence that surgery alone fully accounts for postoperative internal rotation deficit.

At the same time, the absence of clinically relevant GIRD in the conservative group despite a longer duration of sling use suggests that immobilization time alone is unlikely to explain the postoperative pattern observed after ORIF. In this context, surgery-related factors may be relevant. Plate fixation requires dissection along the superior surface of the clavicle, which may affect the surrounding soft tissues, including the functional environment of the upper trapezius. Given the role of the trapezius in scapular stability and retraction, subtle impairment or altered recovery of this muscle may influence scapular positioning after surgery. Although this mechanism was not directly evaluated in the present study, it may partly explain why GIRD in the operative group was accompanied by radiographic and clinical findings suggestive of altered scapular orientation.

Another noteworthy finding is that no significant association was identified between postoperative GIRD and the routinely recorded demographic, injury-related, or peri-treatment variables examined in the ORIF group. This negative result is potentially informative. It suggests that the occurrence of postoperative GIRD may not be adequately explained by conventional clinical variables such as age, follow-up duration, dominant-side involvement, injury mechanism, fracture subtype, duration of sling use, or rehabilitation compliance alone. Instead, more complex mechanisms—including subtle soft-tissue disruption during surgical exposure, alterations in scapular muscle coordination, or postoperative neuromuscular adaptation—may underlie this phenomenon. Further prospective studies incorporating more detailed assessment of peri-clavicular soft tissues, scapular muscle function, and dynamic shoulder biomechanics are warranted.

Accurately quantifying scapular orientation remains challenging. While advanced imaging modalities such as MRI and CT provide detailed anatomical information, their assessment is typically performed in the supine position, which may not reflect functional scapular alignment under the influence of gravity and muscle activation [20, 21]. In the present study, projected area ratios obtained from standard anteroposterior radiographs were used as indirect and exploratory surrogates of scapular orientation in the upright position. In the ORIF cohort, both the G-G ratio and the G-C projected area ratio were significantly higher in patients with GIRD, supporting the potential relevance of radiographic projection changes to the observed kinematic pattern. Importantly, because the G-C ratio can be derived from a single radiograph on the affected side, this parameter may have practical value in settings where bilateral paired imaging is unavailable or undesirable. In this respect, the present findings raise the possibility that a unilateral radiographic surrogate may provide clinically useful information while reducing the need for additional contralateral imaging and radiation exposure. Given the complex three-dimensional motion of the scapula, however, these two-dimensional parameters should not be interpreted as direct or definitive measures of scapular protraction. Rather, they should be viewed as pragmatic, hypothesis-generating markers of altered scapular positioning that warrant further investigation [22]. Future studies incorporating three-dimensional motion analysis [23] or dynamic imaging [24] are required to validate the relationship between these projected area ratios and true scapular kinematics.

Several limitations of the present study should be acknowledged. First, the cross-sectional design precludes evaluation of temporal changes and limits causal inference regarding the relationship between treatment modality, GIRD, and altered scapular positioning. Second, comparison between the operative and conservative groups should be interpreted with caution because treatment allocation was not randomized and baseline fracture severity differed substantially between groups. Third, radiographic assessment was not identical across all study groups. Because contralateral paired radiographs were not available in the conservative cohort, between-group radiographic comparison relied on the G-C ratio rather than a G-G ratio. This limits direct structural equivalence between radiographic measures across analyses. Fourth, the proposed role of surgery-related soft-tissue disturbance, including potential effects on trapezial function, was not directly assessed and therefore remains speculative. Finally, although the findings may have implications for postoperative assessment and rehabilitation, this study did not evaluate specific immobilization or exercise protocols, and no conclusions can be drawn regarding the efficacy of particular rehabilitation strategies.

## Conclusions

GIRD appears to be a common but largely subclinical finding after operative treatment of mid-shaft clavicle fractures. In contrast to the pattern described in overhead athletes, this post-traumatic internal rotation deficit was not accompanied by external rotation gain, suggesting a potentially distinct underlying mechanism. The observed association with a tendency toward scapular protraction may provide insight into this phenomenon. Awareness of this pattern may be relevant to postoperative assessment and may help inform future studies aimed at optimizing rehabilitation strategies.

## Supplementary Information

Below is the link to the electronic supplementary material.

Supplementary Material 1

## Data Availability

The datasets used and/or analysed during the current study are available from the corresponding author on reasonable request.

## Abbreviations

AP: Anteroposterior
CI: Confidence interval
DASH: Disabilities of the arm, shoulder and hand
DR: Digital radiography
ER: External rotation
GIRD: Glenohumeral internal rotation deficit
IQR: Interquartile range
IR: Internal rotation
ORIF: Open reduction and internal fixation
PROMs: Patient-reported outcome measures
ROM: Range of motion
SD: Standard deviation
SICK: Scapular malposition, inferomedial border prominence, coracoid pain and malposition, and dyskinesis of scapular movement
G-C ratio: Ipsilateral glenoid projected area/ipsilateral coracoid projected area
G-G ratio: Affected-side glenoid projected area/contralateral-side glenoid projected area
EARS: Exercise adherence rating scale
