# Supplementary material for: Glenohumeral internal rotation deficit after operative treatment of mid-shaft clavicle fractures: a subclinical, scapula-related phenomenon
Source: J Orthop Surg Res. 2026 Apr 24;21:345. doi: 10.1186/s13018-026-06856-7 (PMC13273958; doi:10.1186/s13018-026-06856-7)
Supplement: Supplementary file 1 — Supplementary Material 1 [file 13018_2026_6856_MOESM1_ESM.pdf]

TABLE 1. *SICK Scapula Rating Scale: Static Measurements: 0–20 Points*

ORTHOPAEDIC SURGERY
MORGAN KALMAN CLINIC
ARTHROSCOPIC SPECIALISTS

DATE ______________________
NAME ______________________
AGE ______________________

SPORT ______________________
POSITION ______________________
PRESENTING SX? ______________________

SUBJECTIVE	PAIN			YES	NO	SCORE
	Coracoid			1	0	
	AC Joint			1	0	
	Periscapular			1	0	
	Prox. Lat. Arm			1	0	
	Radicular			1	0	
OBJECTIVE						
	Coracoid			1	0	
	AC Joint			1	0	
	Sup. Med. Scap. Angle			1	0	
	Impingement Test			1	0	
	Scapular Asst. Test			1	0	
	Tos Paresthesias			1	0	
SCAP. MALPOSITION	0cm	1cm	2cm	3cm	SCORE	
Infera	0	1	2	3		
Lateral Protraction	0	1	1	3		
Abduction	0°	5°	10°	15°		
	0	1	2	3		
		TOTAL SCORE				
